# Academy of Stomatology

**Published:** 1904-01

**Authors:** 

**Affiliations:** Academy of Stomatology


### ACADEMY OF STOMATOLOGY.

A REGULAR meeting of the Academy of Stomatology, of Philadelphia, was held at its rooms, 1731 Chestnut Street, on the evening of Tuesday, April 28, 1903, the President, Dr. R. Hamil D. Swing, in the chair.

A paper was read by Dr. Rodrigues Ottolengui, of New York, entitled "The Great Amalgam Fallacy."

(For Dr. Ottolengui's paper, see page 34.)

### DISCUSSION.

*Dr. William H. Trueman.*—The essayist, in the opening paragraphs of his paper, is solicitous that the discussion shall be strictly confined to the subject presented, and he asks that his position in regard to it shall not be misconstrued. This is his right. The paper is now before us; the various suggestions, deductions, and assertions therein contained, and these alone, are our subjects for discussion. It is important, therefore, that they should be clearly defined, disentangled, and isolated from the rhetoric essential to a well-written scientific essay, in order that we may fully comprehend the ideas expressed and profitably discuss their merits.

This is the more important from the fact that the essayist has, in some measure, departed from the well-beaten track, and has presented his subject in a dress so new that it calls for a discussion on other lines than those with which we have in the past been so familiar. We miss the denunciations of the "nasty stuff," the forceful presentation of its many faults, and the uncomplimentary expressions in reference to those who use it. If I understand him, his plea is for better treatment of this long maligned tooth-filling material. He contends that it is cheap only when cheaply used. He advises that it is well worth while for every operator to thoroughly study the amalgam question with a view to ascertain the best composition, the best method of preparing, and when and how to obtain by its use the very best possible results.

The great amalgam fallacy to which he refers seems to be the use of amalgam by a large percentage of the profession with but little regard to these important points.

Acknowledging, as he does, the intrinsic value of amalgam, his suggestions are directed to giving it a more honorable place in the tooth-saving scheme. As a first step he suggests charging for amalgam fillings a larger fee. Now, it is a question how far a dental society can safely go in discussing this matter of fees. The time was when many dental societies adopted a fee bill, and required their members to observe it as a minimum charge. It did not, however, work well. So many things are to be considered when naming for work like ours a just compensation, that it is difficult, as it is at times injudicious, to attempt to formulate any definite rules. To some dentists, and to some patients, it is merely a matter of education, as the essayist suggests. To many others, however, it is a matter of expediency and ability. Unfortunately, imperative demands upon the earnings of some patients seriously lessen the cash they can command. The question of fees in all its bearings is a problem each operator must settle for himself. No dentist is under obligation to render to a patient a greater service in time and material than the patient is able and willing to pay for, and he is entitled to use his judgment in doing the best he can with the means and opportunity at his command. On the other hand, a dentist is hardly justified in considering his patient a thing to keep appointments promptly, and to promptly meet, without question, any bill that may be rendered. We appreciate and commend the suggestion that if those dentists who can will place upon a well-made and artistically finished amalgam filling the same value that they do upon one equally well done with gold, it will bring this useful material into better repute; it will also bring it more into competition with gold, and will lead to its being much more generally used. It will prove an invitation to both good and evil. Whether it is a wise thing to do is a proper subject for discussion, and as such is presented to you in the paper.

His second point is well taken. It is a fallacy, and a serious one, as he says, to use indiscriminately the many differing compounds included under the word amalgam, without regard to their components, their differing properties, and the special manipulation that these require. I differ with him regarding the remedy. I do not regard the old formula, that of the first alloy placed upon the market and erroneously called "Townsend's," a desirable one to use, notwithstanding that it has been longer and perhaps more generally used than any other. A dentist has a right to know the components of the alloy he uses. He then can ascertain once for all the peculiar properties they confer upon the alloy and upon the resulting amalgam. The market name tells him nothing, the enclosed direction may not be the best for him, and the scientific tests to which it has been subjected before offered for sale were, in all probability, under conditions it will never again meet. I have always made my own. I know what I am using, and am able to trace failings to their cause. There are more things than virtue and merit that keeps these alloys on the market. Properly used in suitable places, the original formula, five parts tin and four parts silver, leaves but little to be desired; nevertheless, it is very far from being a desirable alloy for general use; that it has held its own on the market for half a century or more is due to other causes than intrinsic merit. I would suggest that a dentist, at the outset of his career, make himself familiar with the chemistry and the metallurgy of alloys and amalgams, study closely their properties, learn how to manipulate them and how to make them do him their best service, and insist on knowing the formula of the alloy he adopts in his practice. It is not likely, as the essayist suggests, that the time will soon come when the words "alloy" and "amalgam" will be as definite in their meaning when used by dentists in this connection as is now the word "gold." We have here another thought for discussion.

A third fallacy relates to cavity preparation. Here again the essayist has entered fallow ground. His remarks have, in the main, no relation to the now almost forgottten spheroiding of amalgam. He claims that cavity peparation for amalgam should have at least the same care as that for gold. Aside from mere shaping for retention, he contends that filling-material should not be considered in cavity preparation, that all require the removal of disintegrated and infected tissue, ragged edges, and the same nicety in preparing enamel margins. Owing to the want of edge strength, common to all amalgams, bevelled enamel margins should be avoided. While there are some marked differences in cavity preparation for a material requiring to be welded in position, and one as plastic as amalgam, we can heartily agree with the essayist when he says that it should be carefully, skilfully, and thoroughly done; and he may be right when he adds, "it rarely is."

His further remarks upon shaping and polishing approximal amalgam fillings are worthy of careful attention, and to some extent I can agree with him, indeed, fully in regard to the cases to which he specially refers. It is a high compliment to the tooth-saving qualities of amalgam that it should have attained its present reputation, used and abused as the essayist says it has been. There is room for discussion, whether or not the finish for which he contends is really of practical value, or is merely an embellishment. That it is not generally done goes without saying, some, indeed, contending that the after-polishing is wholly unnecessary and a waste of time. If this is the case, are we justified in putting the patient to the inconvenience and expense which it entails? Are we profitably using our own time and talent? Polishing is necessary when gold is used, because the outer surface of a gold filling is not only rough as left by the plugger, but it consists of imperfectly packed gold which must be removed. Furthermore, in many cases where amalgam proves of much value, the overlaping edges at the cervical border pressing down to the gum tissue, and the large contact surface any attempt to polish would remove, are essential to its best service. This is the case where there has been extensive gum recession and where very large interdental spaces exist. The amalgam, filling this space, tends to cleanliness, and its close adaptation to the surface of the adjoining tooth, where the teeth have become loosened by loss of tooth-supporting tissue, is as important to the patient's comfortable use of the tooth as is its occluding the cavity of decay. The use of amalgam in this way has been criticised as careless and slovenly. When purposely done, with a definite object in view, it is not so; it is then a legitimate and commendable practice. The mere fact that amalgam fillings, thousands of them, have done all the service a filling can do with its contact surface unpolished is very good evidence that it is not a prime essential. There is room here for profitable discussion.

The method of making amalgam inlays does not impress me favorably. It introduces more invitations to failure than it avoids. Lining the cavity with gold-foil is, in some cases, an excellent practice. It does not, however, always prevent discoloration. In some cases the gold remains gold; in others it becomes amalgam, and the tooth discolors more than if it were not there. I find that this is less apt to occur with alloys containing a high percentage of silver than in those in which tin predominates. I find it is not best to be too liberal with the gold. There is danger, if too much is used, that it will not be pressed into close contact with the cavity walls. Why, when gold is thus used in connection with amalgam, the dentine should remain undiscolored, at times for several years, and then, without any apparent cause, discolor, I do not know, but I know it does. Except in the case of frail visible teeth, I see no advantage of a gold lining. It introduces a possible risk of electro-chemical action without any compensating advantage.

Poor amalgam! What a wonderful history it has had!!! The time was, and that not so very long ago, when to confess to its use was professional suicide, and a paper such as has been read to-night would have insured its author a prompt expulsion from his dental society, branded as a quack. We must bear in mind, however, that the amalgam atmosphere of the early forties and that in which Dr. Ottolengui speaks are by no means the same. We now know more about teeth, their surroundings and disorders. Through this has come to pass the great change which has permitted a reputable practitioner to demand that amalgam and gold shall stand side by side, and to merit your applause for so doing.

*Dr. Joseph Head.*—I have been extremely pleased with the conservatism of the paper. The author's method of preparing the walls of the cavity for the amalgam is that which Dr. Flagg taught us,—that is, that the amalgam should be placed at right angles with the lines of mastication. There was one part of the paper which particularly impressed me, because I felt that I belong to that class of dentists who, according to his definition, work in a slovenly way, not taking proper care in polishing the amalgam fillings. When there is no objection to separation, I prepare the cavities with the floor flat. For the sake of argument we will say that I smooth the edges of the cavities. I then take my amalgam, made plastic in a quantity one-half to twice as much as is required to fill the cavity. When the cavity is dried and sterilized I take a small portion of cement and line the margins, squeezing out all of the cement that I possibly can. I then make the edges free from cement. When that is done I let the cement harden, which takes, with the use of hot air, from three to five minutes. When that is done I squeeze the remaining portion of amalgam dry between my fingers and pack it down into both cavities as though one filling. The consistency is that which Dr. Black requires. That is a matter for the individual dentist to decide upon. Dr. Black carefully explains that too great drying of the amalgam is not an advantage. I now have one large mass of filling-materials between two approximal fillings. I then divide the fillings at the cervices, making a V-shaped space almost reaching the occlusal surfaces, and then with a very fine spatula I divide that amalgam into two fillings, being careful, however, that there shall be a good approximation at the top, so there will be no break-down from mastication. To prepare two such cavities will not take the average operator more than twenty minutes. Perhaps it is slovenly work, but I cannot see why, with the polishing which can be done later and in ten minutes, these fillings cannot be made perfectly smooth.

The fillings having been prepared and the gingival margins made accurate, the patient is sent away. When he returns, the amalgam, not being as hard as gold, can be readily polished, and by means of little files the gingival margins can be made perfectly true. When we get the gingival margins perfectly accurate and the sides smooth up to the point of contact we can take a sharp spatula and work it backward and forward, spreading the teeth apart just enough to allow them to spring back again after polishing. I polish them not so smooth as glass, but the polish is perfectly good, inasmuch as the contact point is only of metal. When that is done the polishing can be done on the top. From an experience of five or six years I find this satisfactory for all practical purposes. The time required for filling is only about half an hour. The conductivity of oxyphosphate of zinc depends entirely upon the tooth and the person placing the filling. If it is near a nerve, it may not be a poor conductor, though it is certainly a poorer conductor than gold. Its conductivity of heat is in proportion to the ratio of the conductivity of electricity. While there have been many things said against oxyphosphate of zinc, it has been my experience, and I think that of the profession at large, that it is a good preserver of structure, that it will sustain the tooth from shock, and last as long as any known temporary filling.

I think we are not doing justice to our patients in avoiding an easy method of procedure.

*Dr. Edwin T. Darby.*—When this subject was announced I thought it was going to call forth some pretty spirited discussion, but being privileged to read the paper before it was read to-night, I concluded that Dr. Ottolengui had been so conservative that possibly we should have a tame evening of it, and I am rather inclined to think that he anticipated the same thing, because just before he sat down he threw out a suggestion that I think was intended to warm up the meeting.

I think everything Dr. Ottolengui said in his paper was strictly true. Truth is several-sided, and while everything he said was true, everything that he said might not be expedient. Many things are possible, but they are not expedient. Some of the ideas are perhaps practical, but hardly expedient in all cases. He said that initial cavities in molars were better filled with some other material than amalgam. I think the feeling of the profession at large is that initial cavities in first molars in the mouths of young children are better temporarily filled with amalgam than with gold. I will agree with Dr. Ottolengui when he says that occlusal cavities in first molars are better filled with gold or something else. I believe they are better filled with oxyphosphate of zinc. I feel that by the twelfth or fourteenth year I will have saved these teeth with as little pain as I could have done with anything. I believe, notwithstanding what Dr. Black has said with regard to the hardness of tooth-structure, that clinically the teeth become harder. I know patients become hardened, so that they will bear longer operations at twelve or fourteen than they will at seven or eight.

In regard to the length of time required for filling with amalgam, I think that he is right in the main, and that we could perhaps fill that same tooth with gold and polish it at the same sitting, unless the cavity were too large.

Dr. Ottolengui did not denounce amalgam, as I feared he would. If he would denounce amalgam *per se*, he would meet with a good deal of antagonism from the intelligent body of men before me. It is true that our dental alloys are infinitely better to-day than they were ten years ago. I believe there are better amalgam fillings done to-day than were ever done in amalgam work. I saw fillings to-day which had been done by Bonwill,—four-fifths of them done with alloys. There were four or five beautiful gold fillings. I studied the fillings with respect to the saving qualities of the two materials, and I could not say which was doing the best service. The amalgam work was saving the teeth as perfectly as the gold. I thought then that Dr. Bonwill builded as well as he knew when he used amalgam, and builded as well as any of us know, even though we build with gold. The margins were fine and the contours perfect. That is only one case of many I have seen since he died. His amalgam fillings have been exceedingly well done, and nothing could have saved the teeth better. I am sure that if he were living and here to-night he would emphasize and add very materially to what I have said in respect to amalgam.

With the inlays suggested by Dr. Ottolengui I have never had any experience. I can see that such fillings can be polished out of the mouth, but question the feasibility of removing an amalgam filling that had been thoroughly packed in a matrix of gold. It seems to me it would require great sacrifice of the tooth-structure. It has been said of me that I rush things and have no regard for my patients, but I could not prepare two such cavities in twenty minutes. If I did it well in forty minutes I would think I had worked as rapidly as the good of the patient warranted.

I think Dr. Head's method of separating his alloy after he has packed it is a very good one. That was Dr. Bonwill's method. If we go through the same process with gold, we of course destroy the contour. Dr. Ottolengui has pointed out just the difficulties dependent upon these contour fillings of alloy. It is easier to contour with gold than with plastic material.

I believe that every amalgam filling, no matter where placed should be thoroughly and beautifully polished after it has hardened. Overlapping edges are the cause of many failures in amalgam fillings. They are unscientific, are not beautiful to look at, and they indicate slovenly operators. Amalgam should be trimmed to the margins and finished the same as gold. If this is done I believe it will save the tooth just as long as a good gold filling.

*Dr. John C. Curry.*—I suppose the doctor would say something in regard to the finishing of approximal fillings. The most satisfactory method I have found is to finish the edges when the fillings are about two-thirds inserted, and then with a piece of thin mica placed between the two fillings polish them. I trim off the mica and leave it between the fillings until they are hard. In that way we get absolute contact, so that the natural spring of the teeth will allow the fillings to come together and it is little trouble to finish them. I find that the thin mica can be readily trimmed and left in place of the matrix, and at the end of two hours removed with a piece of dental floss.

*Dr. A. N. Gaylord.*—The first part of Dr. Ottolengui's paper would lead us to believe that very few amalgam fillings were ever inserted that were strictly first-class fillings. I think he has overstepped the mark, for I think that, while there are a great many slovenly fillings put in with amalgam, there are also about as many slovenly gold fillings put in. This is not the fault of the filling, but of the man. In my hands it is not a fact that a properly inserted amalgam filling takes as long as a similarly placed filling of gold. I cannot see why it should be. Amalgam is a plastic material and is placed in the cavity with greater rapidity, and after it is put in the polish is certainly more rapidly done, because of the nature of the material itself. Gold is a metal which cuts very hard and heats up very quickly, whereas amalgam is a more brittle metal which cuts quickly under the disk. After an amalgam filling has been placed it can be nearly accurately shaped by rubbing it with a burnisher and brought down to fair shape and contour. The polishing at subsequent sittings is easily accomplished by strips and disks. I admit that the disk is apt to remove the contour, but by using strips I cannot see any reason why the filling cannot be perfectly contoured and left perfectly smooth. I think there is no poorer filling than a poor gold filling, and because of the more easy adaptability of amalgam, an average filling in the back part of the mouth, accurately placed and polished as best we can, will give better satisfaction than the filling of gold which is not properly placed, and many of them are in such position that it is impossible to give them the benefit of the skill and dexterity desired. A chain is no stronger than its weakest link, and the filling is no better than your ability to properly place it. I have put in many fillings which, if I held the tooth in my hand, I would be mortally ashamed of, but, considering the position of the cavity, I have congratulated myself.

I do not think that the young man can at first charge all his patients by the hour. He has patients who are in moderate circumstances, and those patients must know to some extent what their bill is to be. We must have a scale of prices. It is not the fee that makes the dentist less skilful. It is the dentist's conscience. When it comes to the matter of time charge, which I think is the correct one, I do not know but that we are all day-laborers. I know I feel like one to-night. Where is there a successful dentist who is not a mechanic. Dr. Ottolengui suggests another method, which I think is a dangerous one.

The matter of the amalgam inlay does not strike me so favorably that I could try it in my practice. I cannot see how a man could save any time by making an inlay. You must be able to set that inlay downward. It will mean a very extensive cutting of tooth-structure in order to set such an inlay, and when completed the weakest link is the cement which holds it. The force of the mastication on the molar teeth is such that I do not think it advisable to rely entirely upon cement as anchorage.

I cannot understand how a man with a conscience could subject the little ones who come to us with the inferior first molars defective in the fissures, to the long sitting required for the insertion of a gold filling. The eruption of the tooth in many cases is not sufficient to properly apply the rubber dam without holding it in place with clamps, which would have to be forced down upon the tissues and would cause much pain. I think to handle the little ones in that way is to do them a life-long injury. They grow up with a natural dread because they remember those first few fillings that the dentist put in for them, and every subsequent visit to the dentist is more arduous from a mental stand-point because of the insertion of those fillings. I do not say that amalgam is the best thing, but I do believe in doing anything to relieve the young patient of the tedious sittings required for putting in gold fillings.

*Dr. H. E. Roberts.*—Gold would be the last thing I would put in a child's first molar at seven years of age, particularly as we so frequently first see the child when the cavities have passed beyond the initial stage.

*Dr. E. C. Rice.*—Inasmuch as the essayist has treated amalgam very fairly to-night the remarks I intended to make do not count. I do not think approximal cavities are ever successfully filled with amalgam unless a matrix is well and strongly put in. I do not approve of making fillings after the manner of Dr. Head. I see no objection to the separation of teeth. I do not think an amalgam filling can be polished unless a tooth has been separated. It is just as necessary to make a perfect polish at the point of contact as at any other. Too many amalgam fillings fail because the operator depends upon the sensitivity of the molar rather than upon the conditions of the cavity. A copper matrix can be used which need not be removed at the same sitting. I think amalgam will last just as long and give as good service as gold.

*Dr. J. A. Bolard.*—In the inlay work described by the essayist I have had no experience. It seems to me there would have to be cut away a large portion of tooth-structure, which would be unjustifiable. After cutting away so largely I would prefer to put in something that would look better than amalgam. That amalgam will last without being polished I have evidence in my own mouth. Twelve or fifteen years ago these fillings were put in and never rubbed down. I do believe, however, that they should be polished, and I follow out that idea so far as I can.

I understood from Dr. Ottolengui's paper some time ago at Asbury Park that he never used amalgam. He was criticised at that meeting, and I understood him to say that he never remembered having put in an amalgam filling, and yet in his essay to-night he advocates it when properly employed. If he has been converted I am glad to know it, because I feel there is merit in amalgam. His methods, outside of his inlays, I think are excellent. I am glad if he can get the fees he speaks of; I wish we all could. I do think a man should be remunerated for his services. If we are truly professional men we are not interested in the money alone, but must expect to do for the public a large amount of work for which we get no return. With the paper as a whole I largely agree. If the doctor has had a change of heart it has been in the right direction.

*Dr. Ottolengui* (closing).—Right next to Asbury Park is the place where conversions take place annually. I would say, however, that I am doing less amalgam work than ever. I think the gentleman misunderstood the Asbury Park paper. I did not say I never put in amalgam fillings, for I have put in many of them. What I said was that I did not remember that I had ever put in an amalgam filling on the occlusal surface of a first molar. The paper reads that way, and has not been changed. In regard to Dr. Rice, when that gentleman's discussion is printed, it ought to be printed in all caps; it is the best thing I have ever heard about amalgam fillings. It is so largely in answer to Dr. Head that I need hardly answer that gentleman. I notice a great versatility of talent, for every time I thought I had to answer a remark, the next man speaking answered it for me. I must, however, say to Dr. Head, that the method he suggests is the one by which I lost a tooth in my own mouth, and the work was done by a good dentist too, a dentist who carried out the method to its limit.

I think a very great percentage of the recurrent decay at the cervical margin occurs around amalgam fillings rather than around gold fillings. I do not think it is because it is impossible to polish amalgam there, but simply that it is not polished there. That the amalgam can be made over when one-half set is one of the greatest errors. In trying to make specimens of inlays I have necessarily done work out of the mouth, and I would like some of you gentlemen to try it for experience, and you will find one error I did not point out. You will find that amalgam is not as plastic as you are apt to think. You could adapt gold to rough edges better than amalgam. You may find that your alloys are in suspension in the mercury, and in forcing them out you force out a lot of mercury and leave some rough margins, and then in putting in the amalgam you never afterwards can get a polish. The little particles chip off and you will have crevices. The gold put in in not too great pieces has a plasticity that the amalgam does not have.

The matter of the inlays I suggested as worthy of trial. Since the discussion I do not think it so worthy. If, however, you do not try it in the mouth, it would be well to try it extra-orally. If Dr. Head would take two teeth with such cavities as he describes, put them in plaster of Paris, file and polish them in the way he has advocated, and split them open, he will find them less well polished than he thinks.

In putting the inlays in you do not have to cut away as much as you imagine. If you desire to have some of the deeper cuts left so that you will not force your matrix in, you could simply fill those places first with cement. Amalgam fillings should always have cement between them and the tooth. I have great faith that fillings of any kind with cement so placed will defy decay longer than any other fillings put in teeth.

I tell my friends in Brooklyn, when they speak in the same way about the cruelty of filling young people's teeth with gold, that I would be glad to take the children off their hands whom they are averse to taking care of, but that I would fill the teeth with gold instead of with amalgam. The only part of my practice in which I place absolute reliance are the grown up people in whose mouths I put gold when they were children. I do not recall having lost a child for whom I filled teeth, except by their having moved from the city or through their marriage. The fact that I have thus retained them is due to the fact that the very time to fill the teeth with the least pain is before they have decayed. The object in putting in gold is that I can prepare the sulci at that time smaller than at any other, and the sulci being prepared smaller, I am safer with the gold filling than with the amalgam, for it is my experience that the cavities can be filled painlessly. You might have a child shrink, and in that case put in gutta-percha and wait. The fact that some teeth are sensitive does not militate against the practice. In those cases I fill with gutta-percha, and in them I ask you to avoid amalgam. The public has been educated to the fact that amalgam is a permanent filling. If you put in gutta-percha, not only does the patient understand that that is temporary, but they are obliged to come back. A good filling can be put in for children of eight years of age and less, and when put in that is the end of it. That tooth never requires to be filled in that place again, and that in itself is a sufficient reason for the practice. It is not because I do it that I advocate the method, because I can name hundreds of men who fill teeth a thousand times better than I do. To get the same effect with amalgam as is secured with gold in these fillings requires greater destruction of tooth-structure, weakening of the walls, enlarging of cavities unprotected by enamel, and in my opinion this invites disaster.

After a vote of thanks to the essayist the meeting adjourned.

Otto E. Inglis,
*Editor Academy of Stomatology.*

